# Neighborhood Contexts and Oral Health Outcomes in a Pediatric Population: An Exploratory Study

**DOI:** 10.3390/children8080653

**Published:** 2021-07-29

**Authors:** Vladyslav A. Podskalniy, Sharat Chandra Pani, Jinhyung Lee, Liliani Aires Candido Vieira, Hiran Perinpanayagam

**Affiliations:** 1 Schulich School of Medicine & Dentistry, University of Western Ontario, London, ON N6A 5C1, Canada; vpodskal@uwo.ca (V.A.P.); lvieira9@uwo.ca (L.A.C.V.); hperinpa@uwo.ca (H.P.); 2 Department of Geography and Environment, Faculty of Social Science, University of Western Ontario, London, ON N6G 2V4, Canada; jinhyung.lee@uwo.ca

**Keywords:** dental treatment outcomes, geographic information system (GIS), neighborhood contexts

## Abstract

Aims: This study aimed to explore the impacts of neighborhood-level socioeconomic contexts on the therapeutic and preventative dental quality outcome of children under 16 years. Materials and Methods: Anonymized billing data of 842 patients reporting to a university children’s dental over three years (March 2017–2020) met the inclusion criteria. Their access to care (OEV-CH-A), topical fluoride application (TFL-CH-A) and dental treatment burden (TRT-CH-A) were determined by dental quality alliance (DQA) criteria. The three oral health variables were aggregated at the neighborhood level and analyzed with Canadian census data. Their partial postal code (FSA) was chosen as a neighborhood spatial unit and maps were created to visualize neighborhood-level differences. Results: The individual-level regression models showed significant negative associations between OEV-CH-A (*p* = 0.027) and TFL-CH-A (*p* = 0.001) and the cost of dental care. While there was no significant association between neighborhood-level sociodemographic variables and OEV-CH-A, TRT-CH-A showed a significant negative association at the neighborhood level with median household income and significant positive association with percentage of non-official first language (English or French) speakers. Conclusion: Initial analysis suggests differences exist in dental outcomes according to neighborhood-level sociodemographic variables, even when access to dental care is similar.

## 1. Introduction

Dental caries is one of the most common childhood diseases and the most common oral disease in children. The Canadian Health Measures Survey (CHMS) estimates that dental caries affects nearly 60% of all Canadian children [1,2]. In recent years, there has been increased interest from both clinicians and researchers in the role of socioeconomic and demographic contexts in the oral health of children [3,4,5]. There is growing evidence that sociodemographic variables such as family income and education strongly correlate to dental caries in children [6,7,8]. This has led to the development of several theoretical models for caries risk prediction based on social and demographic variables [9,10,11]. 

The postal code, as a descriptive unit of a neighborhood, has been effectively used to measure the spatial impacts of health data [12]. In Canada, the first three digits of the postal code are called the Forward Sortation Area (FSA) code, a spatial unit that allows for the collection of demographic data, without revealing the identity of individual families in the area. Statistics Canada collects neighborhood-level data on several demographic factors, including but not limited to education, household income and immigration status FSA code as a spatial unit of measurement. While some efforts have been made to estimate the distribution of dental caries, there have been few attempts to explore the association between neighborhood factors and children’s oral health outcomes in Canada [13,14,15]. 

The concept of utilizing secondary data from patient records is not new. However, advances in technology, computing power, and the ease of access to digital records have resulted in rapid strides in the fields of data mining and knowledge discovery in database (KDD) [16]. In the United States, the data mining of electronic health records and billing data has been shown to serve as an accurate indicator of both risk of caries and access to dental care [17]. The dental quality alliance (DQA) has proposed definite protocols to mine electronic dental records to identify the quality of care received by specific dental communities [16,17]. These quality outcomes can be used to assess the access to dental care (OEV-CH-A), utilization of preventative dental services such as topical fluoride for the prevention of caries (TFA-CH-A) and/or the utilization of therapeutic dental services (TRT-CH-A). Most dental care in Canada is provided by private dental practitioners [1] and though billing practices differ according setting and province, the suggested fee for the procedures performed is coded and these codes remain largely uniform across a province. This relatively uniform coding system allows for the comparison of billing data from different practices and settings.

The adoption of electronic databases and billing systems by dental schools across North America has resulted in an increased capacity to mine these patient records and billing data [16]. The Children’s Dental Clinic at Schulich Dentistry has offered subsidized oral health care to the children (below 16 years of age) of London and the surrounding areas of Southwestern Ontario for over four decades. Since 2011, the clinics have adopted an electronic billing system, which documents all treatment rendered at the Children’s Dental Clinic using treatment codes proposed by the Ontario Dental Association (ODA). 

Geographic Information System (GIS) enables gathering, managing, visualizing, and analyzing neighborhood socioeconomic, demographic as well as health outcomes data in an integrated framework [10,18]. There are several methods of spatial analysis and GIS, which have been used with varying degrees of success in dentistry [19]. The major use of GIS techniques in dentistry has been in visualizing the spatial distribution of disease [20].

This study aimed to measure the impacts of neighborhood-level sociodemographic data, as well as individual-level cost, on the therapeutic and preventative dental quality outcomes of children aged 3 to 16 years residing within the London Metropolitan Area (LMA) of Southwest Ontario, Canada.

## 2. Methodology

### 2.1. Ethics Approval

Approval was obtained from the Health Sciences Research Ethics Board (HSREB), Western University (ID-115567) to access anonymized billing data of patients reporting to the Children’s Dental Clinic of the Schulich School of Medicine and Dentistry between March 2017 and March 2020. Consent for the use of anonymized data for research purposes was provided by the parents/guardians of all children who visited the clinic.

### 2.2. Screening of Patient Records

The patient billings from the electronic billing data from the Children’s Dental Clinic of the Schulich School of Medicine and Dentistry from March 2017 to March 2020 were exported into anonymized Microsoft excel spreadsheets. Records of patients aged below 16 years at the time of the last dental visit were screened for the following inclusion criteria:

(a) Presence of key patient attendance metrics including, treatments, age, date of treatment, ODA treatment code, FSA code;
(b) Presence of at least one additional treatment code within a 180-day period, intended to avoid skew or bias, in keeping with the protocol set by the DQA [14];
(c) At least 20 patients in each FSA code to ensure adequate weight for the neighborhood-level data.

### 2.3. Measurement of Dental Quality Outcomes

Three outcome measures were chosen from the DQA outcome measures. Treatment Services (TRT-CH-A) was described as the percentage of patients who had received a treatment procedure in a one-year interval [16]. Oral Evaluation (OEV-CH-A) was described as the percentage of children who had at least one scheduled oral examination in a year [16]. Topical Fluoride for Children at Elevated Caries Risk (TFL-CH-A) was described as the percentage of children with at least two topical fluoride applications [16].

### 2.4. Data Coding, Analyses, and Mapping

**Figure 1:**
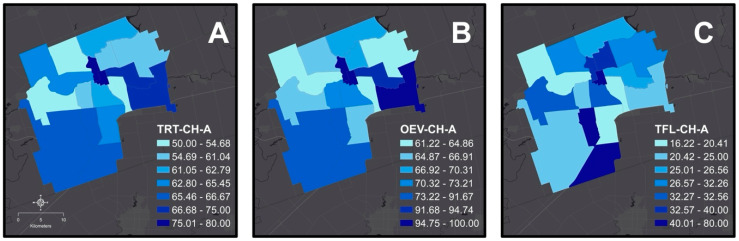
Geographic patterns of dental outcomes at FSA level: (**A**) Treatment received, (**B**) Visits to the dentist, and (**C**) Topical Fluoride Application.

The three DQA outcome variables, treatment cost according to the ODA fee guide and the subsidized fee paid at the dental school by each patient were entered into a single spreadsheet (Microsoft Excel, Microsoft Corp. Palo Alto CA). In accordance with the ethics protocol and to maintain patient anonymity, FSA code information (rather than full residential address data) was obtained directly from patient records, and the resulting neighborhood dental outcomes were visualized using a GIS software (ArcGIS 10.8.1, ESRI Canada, Toronto, ON, Canada). Figure 1A–C presents the spatial patterns of TRT-CH-A, OEV-CH-A, and TFL-CH-A, respectively.

An individual-level linear regression model using the cost of dental care (both subsidized and recommended cost) and DQA measures as factors was developed. Binomial logistic regression models were developed using the presence or absence of each DQA outcome measure as the dependent variable and neighborhood-level household income, percentage of households with college education and percentage of households recently immigrated to Canada as factors. A post-hoc power estimation was carried out to determine the potential number of FSA codes needed to generate a valid FSA code level regression in future studies. Regression modelling was performed using the IBM-SPSS ver.25 data-processing software (IBM Corp., Armonk, NY, USA), while the post-hoc power was calculated using the G-Power sample power calculator (Universtat-Kiel, Kiel, Germany). 

## 3. Results

Data from a total of 17 of the 23 FSA codes in the London Metropolitan Area met the inclusion criteria. A total of 842 patients (*n* = 842) were included, with a mean age of 9.5 years (SD ± 3.6). The population subset included 436 males (mean age 9.4 years, SD ± 3.3) and 406 females (mean age 9.5 years SD ± 3.4). ODA fees were calculated for each patient and the mean cost of treatment was $53.2 (SD ± 40.5). The mean subsidized fee paid by the patients was $17.2 (SD ± 12.2). The DQA outcomes and cost of treatment was described for each FSA code (Table 1). 

Individual-level linear regression with median income as the dependent variable found that individual-level regression modelling showed that the utilization of treatment (TRT-CH-A) did not have a significant association with the cost of treatment when either the cost of treatment or subsidized treatment were used as dependent variables (Table 2). In contrast, both oral evaluation (β = −0.110, *p* = 0.027) and topical fluoride application (β = −0.177, *p* = 0.001) had a significant negative association with cost of treatment when the suggested fee guide was used. The associations remained valid even when the subsidized value was used as the dependent variable (Table 2).

Binary logistic regressions for the impact of FSA code level demographic characteristics on the presence or absence of treatment outcome variables found no significant association between access to care (OEV-CH-A) and any of the neighborhood-level demographic variables measured. When the presence of a treatment (TRT-CH-A) was used as a demographic variable, it was found that there was a significant negative association between median household income and a significant positive association with percentage of the population that spoke a non-official first language and number of children who needed a therapeutic dental intervention in the last year. When preventative care was used as the dependent variable, the associations were reversed with a significant positive association with the presence of a preventative measure and negative association with the percentage of population that spoke a non-official mother tongue. Percentages of individuals with less than a secondary school education, recent immigrants and visible minorities had no significant associations to the outcome variables measured (Table 3). 

A post-hoc power estimation of the number of FSA codes showed that 17 FSA codes with an odds ratio of 2.5, H0 of 0.5 and alpha of 0.05 gave a power of 0.420 for the current sample. It was estimated that, based on this initial data, a minimum of 67 FSA codes would be needed to create a model with a power of 0.95.

## 4. Discussion

The role of neighborhood socioeconomic and demographic contexts on oral health and dental care outcomes have been recognized in the dental literature. One of the greatest advantages of neighborhood-level analysis is that it helps policy makers visualize the difference between individual needs and community needs [19]. There have been recent attempts to geographically examine dental outcomes, and while studies in the United States have managed to use point-level data to demonstrate spatial correlations [21], the fact that this study was using retrospective data meant that ethics concerns only allowed the area level mining of data using FSA codes. The FSA code is also the area level variable at which census data in Canada are shared publicly. The purpose of this study was to visualize dental treatment outcomes at the FSA code level and explore if the patterns that arose were related to the available socioeconomic data collected by Statistics Canada.

There is evidence to suggest that patient access to oral health care and the cost of dental treatment are associated with sociodemographic variables [22,23,24]. There is also evidence to suggest that children from families with poorer sociodemographic indicators tend to have a greater need for therapeutic dental care, and less access to preventative dental treatments such as topical fluoride therapy [5]. It was for this reason that the study chose to focus on the access to dental care (OEV-CH-A), treatment burden of dental disease (TRT-CH-A) and regular preventative care in the form of topical fluoride application (TFL-CH-A). The decision to use the data from the clinics of the dental schools was based on the fact that the school uses a pay-for-treatment model. Even though the treatment is subsidized, the care is utilized by children across the London Metropolitan Area. Another reason for choosing data from the University Clinic was the fact that there is no uniform public database on dental billing across practices in a province where the majority of dental treatment is provided by private dental practitioners [2].

While the cost of dental care is often cited as a barrier to access to dental care [3,5], the current study found that there were no neighborhood-level associations between the socioeconomic variables measured and the access to dental care. This could be explained by the subsidized nature of dental fees charged in the dental school clinic combined with government-funded therapeutic and preventative oral healthcare for children from low-income households in Ontario. However, despite this apparent access to care, there were significant differences in treatment outcomes when neighborhood-level sociodemographic variables were assessed. This in keeping with Medicaid data from the United States, which have shown that even when subsidized healthcare is offered, sociodemographic variables often influence the actual utilization of care [21]. The current dataset was limited to the dental clinics of a single teaching hospital and must be viewed within their limitations. However the initial results suggest that the patterns generated by this analysis are similar to what has been observed at the individual level in previous studies in Canada [25,26]. This seems to suggest that a greater availability and analysis of province-wide, or indeed nationwide, billing data can provide useful insights into the impacts of social and economic variables on dental healthcare outcomes in Canadian children.

The results of the treatment burden showed that, at the neighborhood-level, the burden of dental treatment (TRT-CH-A) was inversely associated the median income of the family, while the utilization of preventative services (TFL-CH-A) was positively associated. This seems to suggest that not only is the burden of treatment in oral disease higher for households with a lower income [27,28], but this may be generalizable at a neighborhood-level as well. This is particularly important as income is used as a screening criterion for eligibility to enroll in subsidized oral healthcare programs in Canada [13]. However, there was also a significant positive association between the burden of dental treatment (TRT-CH-A) and the percentage of individuals in a neighborhood that speak a non-official first language. This is in keeping with the literature that suggests that income inequality and inequity alone are not clear indicators of oral health discrepancy and disparity, and that there are social and cultural determinants of oral health that are often overlooked [24]. 

Preventative dental outcomes are widely understood to better the oral health of patients [29,30]. The fact that TFL-CH-A was inversely associated with the overall cost of dental care supports the literature that shows that regular preventative dental care can lower overall dental treatment costs [28,31,32]. TFL-CH-A was negatively associated with median household income, which seems to suggest that even where access to care is not a factor, patients from neighborhoods that have a lower median household income have a lower utilization of preventative dental services. However, the fact that this pattern was also true of neighborhoods where there was a greater percentage of the population speaking a non-official first language seems to indicate that there may be cultural aspects to the utilization of preventative dental care that need to be investigated further.

Data gleaned from the mining of secondary sources are not a substitute for purposefully collected primary data. However, considering that the last nationwide oral health survey in Canada was conducted in 2008 [1,2], there is a need for a robust network of secondary data. The results of this pilot study must be interpreted with caution, and should be viewed keeping in mind certain limitations. The fact that the study was conducted over a relatively small area means that, while the insights offered by this pilot study are useful, they are not generalizable. The study only measured a sample reporting to a subsidized dental clinic, which meant a significantly smaller sample than those studied in the analysis of insurance or program-level data. The relative size of the population meant that there was not enough data to run multi-level models, which could offer valuable insights into the nature of these neighborhood-level associations. The incorporation of more units (FSA codes) would also allow for accurate spatial regression models such as spatial lag/error models, as well as the geographically weighted regression (GWR) technique, which could then explain the nature of these neighborhood-level associations, taking spatial autocorrelation effects and geographically varying relationships between a dependent variable and covariates into account. Furthermore, the need for patient anonymity meant that data could only be visualized at the level of the FSA code, which did not allow for point-level analysis. This, however, must be viewed in light of the fact that Statistics Canada does not make public or allow for the mining of individual-level data. Post hoc tests of our sample show that valid multilevel models could be created with data from as few as 67 FSA codes. Despite these limitations, the results of our exploratory study suggest that neighborhood-level outcomes of dental care may offer deeper insight into the impact of socio-economic and demographic variables on the dental outcomes of Canadian children. There is a need for studies using a larger number of FSA codes to explore the impacts of different socioeconomic and demographic variables documented through census data in Canada on dental treatment outcomes in children.

## 5. Conclusions

Within the limitations of this study, we can conclude that, even when the access to care is similar, neighborhood-level variations appear to exist in the dental outcomes of Canadian children. These variations might be influenced by socio-demographic variables, which need further analysis to explore the impacts that economic and social determinants such as median family income, language spoken at home and the potential role of cultural factors may have on dental healthcare outcomes.

## Figures and Tables

**Table 1:** Descriptive Statistics and DQA outcomes of the population.

FSA Code	Number of Patients	Oral Evaluation (OEV-CH-A) (%)	Topical Fluoride Application (TFL-CH-A) (%)	Treatment Utilization (TRT-CH-A) (%)	Average Cost Per Patient ($)
N5H	32	94	6	78	200
N5P	28	87	7	53	110
N5R	21	93	57	64	124
N5V	31	65	32	55	113
N5W	24	95	26	74	194
N5X	64	70	27	63	142
N5Y	77	70	34	61	123
N5Z	49	61	20	53	104
N6A	21	100	40	80	84
N6B	22	100	80	80	163
N6C	43	72	33	63	152
N6E	55	65	20	65	114
N6G	179	67	27	55	129
N6H	74	65	16	65	155
N6J	56	73	25	61	135
N6K	44	65	33	50	168
N6L	22	89	78	67	179

**Table 2:** Individual-level association between outcome measures and cost of treatment and median household income.

Dependent Variable		Unstandardized Coefficients *		Standardized Coefficients *	*t*	Sig.
		B	Std. Error	Beta		
Cost of Treatment (ODA Fee)	TRT-CH-A	1.477	1.522	0.044	0.971	0.332
	OEV-CH-A	−5.712	2.580	−0.110	−2.214	0.027 **
	TFL-CH-A	−4.714	1.362	−0.177	−3.461	0.001 **
Cost of Treatment (Subsidized Fee)	TRT-CH-A	1.638	1.498	0.049	1.093	0.275
	OEV-CH-A	−4.235	2.396	−0.095	−1.951	0.035 **
	TFL-CH-A	−4.086	1.213	−0.154	−3.368	0.001 **

* Calculated using linear regression modelling. ** Associations Significant (*p* < 0.05).

**Table 3:** Impact of neighborhood (FSA)-level demographic variables on the presence or absence of dental treatment outcome variables in the sample.

		Access to Dental Care (OEV-CH) (*n* = 842)		Therapeutic Needs (TRT-CH) (*n* = 842)		Preventative Care (TFL-CH) (*n* = 842)	
		B *	Sig.	Sig.	B *	B *	Sig.
Neighborhood-Level Variables ^a^	Median Household Income	0.320	0.358	−0.609	0.012 **	0.646	0.017 **
	% of Non-official First Language (English/French) Speakers	−0.318	0.499	0.660	0.002 **	−0.627	0.029 **
	% of Population with less than Secondary School Education	−0.514	0.170	0.397	0.153	−0.426	0.229
	% of Population who were recent immigrants to Canada	0.580	0.218	0.294	0.345	−0.286	0.457
	% of Population who are visible Minorities	−1.070	0.070	−0.310	0.446	0.036	0.945
	Constant (model validity)	1.890	0.000	−0.482	0.000	1.467	0.000

^a^: Calculated using average FSA code level data from the Statistics Canada Database. * Calculated using a binary logistic regression for presence or absence of each DQA outcome in an individual. ** Associations significant at *p* < 0.05.

## Data Availability

Data will be made available upon reasonable request to the authors.
